# Awareness and performance of blood transfusion standards in operating rooms of Shiraz hospitals in 2012 

**Published:** 2015-04-20

**Authors:** R Robati, E Mirahmadi Nejad

**Affiliations:** **1.Department of Microbiology, Shiraz Branch, Islamic Azad University, Shiraz, Iran.**

**Keywords:** Blood Transfusion, Hospitals, Operating Rooms, Reference Standards

## Abstract

**Background:**

Assuring safety and survival of blood in vitro depends on anti-coagulation substances, blood bag characteristics, storage conditions, and transport of blood. Besides, careful selection and screening of donors as well as blood tests can minimize the transmission risk of blood-transmissible pathogens and optimize blood health. The aim of this study was to assay the level of knowledge and practices among anesthesia technicians on blood transfusion standards.

**Materials and Methods:**

This descriptive cross-sectional study was performed among 85 anesthesia technicians Shiraz, Iran throughout 2012 who were examined by census using blood transfusion questionnaires and checklists. The data were analyzed using SPSS 16 software.

**Results:**

The obtained findings indicated that 32.44% of the technicians have corrected knowledge of blood transfusion standards; nevertheless, 73.84% have corrected performance.

**Conclusions:**

The technicians mostly performed based on their habit and experience. However, their knowledge about blood transfusion and blood bag storage was low.

## Introduction

Identifying the blood group system by Karl *Landsteiner* and his colleagues, and breaking out of World War II, blood transfusion became common (1). Assuring survival and quality of blood and blood products including blood condition *in vivo* is a complex process depending on the formulation of anticoagulant substances, blood bag properties, storage conditions, temperature, and blood bag transport. Therefore, an attempt should be made to provide a suitable environment for survival and metabolism of red blood cells in vitro (2).

Today, most people who are in need of blood transfusion and blood products are patients who are under treatment because of bleeding, anemia resulting from chronic diseases, blood coagulation disorders, platelet problems, and other similar cases (3). Blood is considered as an ideal environment for transmitting infectious agents and is dangerous to human health. Viral, bacterial, parasitic, and fungal agents use this environment to feed and disseminate themselves to other parts of the body and various tissues, thus, lead to extremely lethal and dangerous diseases which eventually cause disability or death of the blood recipient. Therefore, due to the difficulty and high cost of healthy blood supply as well as numerous side effects resulting from blood and its products for patients who are in need of it, and in view of the problems associated with unsafe and untimely blood transfusion that can cause the spread of fatal diseases such as AIDS and hepatitis, it is necessary to consume it on the basis of standard principles and criteria, and actual needs of patients (4). Non-hemolytic transfusion reactions such as non-hemolytic febrile reactions, allergic reactions, intravascular hemolysis, bacterial infection, cardiovascular complications, reduced body temperature, and citrate toxicity are also possible to occur by blood transfusion (6). Factors such as the amount of information and knowledge, education and proper training and experience of the blood transfusions technician play a keyrole in correct and standard method of blood transfusion. Therefore, timely patients' detection need or lack of need to blood transfusion during surgery, owing to the risks that either injection or lack of injection have for threatening the patient's life, is one of the foundational tasks that is usually required for the technicians to know. They should also know blood bag storage and the conditions required storing it in operating rooms, and use blood and its products based on standard and scientific principles and actual needs of patients. The present study was carried out in Iran for the first time to study scientific principles and standards of blood transfusion in operating rooms. The aim was to explore the strengths and weaknesses of transfusion standards in operating rooms so we come to know whether in treatment centers, blood storage and administration is performed based on scientific principles, and if necessary, to establish instruction for training or retraining of the operating room technicians.

## Materials and Methods

This study was Cross-sectional which included 85 anesthesia technicians of operating rooms in hospitals in Shiraz who were responsible for the care, control, and maintenance of anesthetized patients, and if necessary, transfuse blood to the patients during surgery through 2012. Of these staff, 49 were female and 36 were male and all of them were surveyed by census. In this approach, the technicians’ performance from blood delivery from the blood bank laboratory or a specific department to preparation and to the end of the administration was checked by a standard checklist prepared for this purpose. The researchers did their best to have a subtle control over the technicians’ performance to avoid their behavioral changes and performances. The content validity index and reliability of the questionnaire were calculated by using the Lawshe's method and the Cronbach's alpha ratio and the results were 0/76 and 0/78, respectively. In this method, the technicians’ performance from blood delivery to preparation and to administration were controlled and marked in the checklist (as correctly or incorrectly) based on the principles of blood transfusion (according to the standards set by the Iranian Blood Transfusion and Blood Transfusion Medicine) after observing their behaviors performances. In order to assess each technician's knowledge of storage standards and blood transfusion after the end of the administration, she/he was interviewed and responded some questions related to calculating blood volume and blood loss, blood bag storage conditions, and so forth, and her/his responses were recorded in the answer sheet. The papers were then collected and after receiving specific values and data extraction by descriptive statistics analysis using SPSS software 16 were subjected to data analysis. To determine the level of technicians’ awareness based on their responses to questions about the calculation of the volume of blood lost, blood bag storage conditions, storage and blood transfusion standards after the administration, a score within the range of 0-20 was given, more specifically, 0-5 for irrelevant or incorrect responses, 6-10 for poor and incomplete responses, 10-15 for average responses, and 16-20 for complete and good responses was considered. After collecting data, as none of the technicians had received a score in the range of 0-5, the following valuation method was proposed: 6-10 as low, 11-15 as medium and 16-20 as high responses.

## Results

The results of this study indicated that the population was comprised of 57/6% female, and 42.4% male technicians of which 39% were formal employment force and 61% were contract employees. In this study, 28.2% of the population had BA degree (14 females, 10 males), 54.1% had Associate’s degree in anesthesia (34 females, 12 males), and 41.1% were high school graduates who were referred to as experimental technicians (25 females, 15 males). The anesthesia technicians had (%100) correct performance in relation to checking the characteristics of the blood receiver with blood bags as well as selecting proper angiocath and vessel since these are among the most basic principles of blood transfusion instruction and suggest that authorities had been more sensitive in implementing these principle. But, with regard to monitoring patient's vital signs as an important point during injection, only 73.84% were functioning correctly (Table I). Concerning the timely return of the extra blood or the whole blood if it is not needed, which is also of high importance, only %34/1 functioned properly and 65.9% had a totally incorrect performance. In view of the obtained results in this study, %45.9 of the anesthetic technicians were able to diagnose whether or not a patient was in need of blood (Table II).

In addition, since during operation, the patient is unconscious and the early signs of blood incompatibility and protective reflexes are undermined by anesthetic drugs and are hard to diagnose, lack of knowledge about these symptoms can result in serious and uncompensable damage in the patient. As it was observed, %71/8 of the population did not have awareness of the incompatible symptoms of blood transfusion during anesthesia and %67.1 of the technicians did not consider the initial rate of blood transfusion during surgery in emergency situations which, in turn, may cause severe complications. Based on the research findings, not only a small number (%9.8) of anesthesia technicians had sufficient knowledge of blood storage and conditions, but some blood bags might also lose their physiological efficiency during injection or while being returned to the blood bank and contain developed or active infectious agents (Table II).

**Table I:** Absolute and relative frequency distribution of anesthesia technicians’ proper performance based on the principles and standards of blood transfusion in Shiraz hospitals

	**Cases of observing blood transfusion based on principles and standards**	**Correct performance **		**Incorrect performance**	
		**No.**	**%**	**No.**	**%**
1	Timely removal of blood from the fridge for injection	79	92/9	6	7/1
2	Systematic heating of the blood	75	88/2	10	11/8
3	Considering the time interval from putting the blood out of the fridge to injections	41	48/2	44	51/8
4	Controlling patient characteristics with the blood bag	85	100	0	0
5	Proper selection of angiocath and vessel	85	100	0	0
6	Observing the initial rate of injection	28	32/9	57	67/1
7	Method of diluting blood bags	78	91/7	7	8/3
8	Monitoring patient's vital signs every 5 minutes at the beginning of the injection	47	55/2	38	44/8
9	Lack of blood transfusion by other serums from a IV line	81	95/2	4	4/8
10	Returning the unused blood to a blood bank	29	34/1	56	65/9
	Average performance	63	73/84	22	26/16

**Table II:** Absolute and relative frequency distribution of the level of technicians’ awareness of the principles and standards of injection and storage of blood bags in Shiraz hospitals

	**Awareness about blood transfusion standards**	**Degree of correct awareness based on standards **		**Degree of incorrect or insufficient awareness**	
		**No.**	%	**No.**	%
1	Diagnosing patient’s need for blood	46	54/1	39	45/9
2	Familiarity with blood volume formulation at different ages	28	32/9	57	67/1
3	Familiarity with calculating acceptable bleeding volume formulation	32	37/6	53	62/4
4	Familiarity with blood bag storage in operating room	8	9/4	77	90/6
5	Familiarity with signs of incompatible blood during anesthesia	24	28/2	61	71/8
	Average awareness of anesthesia technicians	28	32/44	57	67/56

## Discussion

Blood transfusion for most people who are in need of this precious fluid during different stages of a surgery has always been associated with anxiety and fear. Fear of transmission of blood-transmittable diseases, allergic reactions resulting from the infusion of alien blood, blood transfusion of a wrong blood group or the one which has not been stored or transported according to standard principles are some examples (7). Improper transport of nutrient and waste materials in the body and protection of the body against invading microorganisms puts the balanced and normal procedure of the body in danger. The cardiovascular (circulatory) and lymphatic systems in the body are in charge of performing these vital functions in the body, and blood is the primary substrate of this transportation. Blood is the only tissue circulating in the body and one of its critical functions is delivering materials to the cells and removing waste materials from them. In addition, it provides many tools necessary for coping with outside pathogens, and the does the body's internal adjustment (8). Supplying blood and blood products, the tests performed on them, preparing, storing, transportation, and administration each has its own particular process.

This process should retain the power and purity of the final product, minimize contamination and microbial proliferation, and prevent or minimize physical and chemical changes during blood storage. Today, blood transfusion is considered as an important part of the healing process and like other medical interventions it may be associated with some complications. Providing maximum health and reducing complications resulted from blood transfusions in patients may depend on various factors. One of the standards of maintenance and distribution of blood as a biological product is to store it under controlled temperature. Since the products removed from the blood bank are unstable, blood products which are prepared in a certain temperature also require special temperature conditions during transportation, storage, and use; otherwise, there will be a risk of bacterial contamination. Low temperature may also be risky. Products that require low temperature including whole blood, packed red blood cells, plasma and cryo, and particularly whole blood and packed red blood cells which require a temperature of 1-6° C, should not be kept outside the fridge more than 30 minutes; otherwise, they may be hazardous for patients (9). So, if properly used, blood can be life-giving but, as it is evident in some cases, blood and blood transfusion products are often used inappropriately, placing the recipients at serious hazards (6). Blood and its products have a limited lifetime. To have an optimized use of these products within this time limit, it is necessary to be aware of standard blood storage, transportation, and transfusion principles as well as their side effects (10). Several people in hospital are involved in production and blood and blood administration products from the blood bank to the patient’s bed, and also the probable return of these products (in case it is not used). Nurses and anesthesia technicians are among the main people responsible for this cycle in hospitals. The scientific supervision of this process would be useful provided that these technicians have scientific knowledge necessary for storage and blood transfusion related to their responsibilities. 

As noted earlier, most of anesthesiologists merely rely on the patient's clinical status reported by anesthesia technicians, leaving them to decide whether or not a patient needs blood transfusion. This issue is of high importance because the possible follow-up complications are not controlled and followed in patients receiving blood after hospital discharge and follow-up cannot be controlled. The findings showed that 67/56% of anesthesia technicians were not familiar with standard formula of acceptable bleeding volume with a high confidence coefficient for diagnosing patient's need for blood during surgery. This shows the necessity of reviewing and practicing the required trainings in this area (Table II).

However, given that most of anesthesia technicians do not have sufficient knowledge of storage condition, and blood transportation chain before its use in clinical practice and have mainly acquired their knowledge experimentally, it is necessary to consider instruction of managing the process of blood storage and use as well as the health of blood and blood products for anesthesiology students in educational institutions. Authorities in the field also need to think about the inclusion of materials related to blood transfusion science to academic centers and employ experienced instructors in this field or cooperate with their colleagues in blood transfusion centers to create and increase the existing knowledge about blood transfusion medicine. Results were similar to Asadi Fakhr and his colleagues which was done in Hamedan province (11). Most investigations have focused on possible causes of fever produced by blood transfusion in patients receiving blood, and blood transfusion complications stemming from negligence in the storage method, transportation and other side effects have rarely been studied. Thus, lack of standards for blood storage, transportation, preparation, and key points during a blood transfusion, not only prolongs the recovery time and hospital stay but also, may lead to patient’s death.

In a study carried out from 1996 to 1999 in Brazil on 164 patients who had received blood during heart surgery, it was found that after 6 months, one of these patient got hepatitis type C (12). The results of another study conducted in India indicated that factors such as hepatitis B and C, HIV, malaria, syphilis, cytomegalovirus, parvovirus, and bacterial infections are the agents that can be transmitted to blood receivers. HIV prevalence in different countries through blood transfer from one country to another is different under the influence of various factors. For example, in India in every 1000 blood donors, %0/2 have been proved to have HIV (13, 14).

In another study conducted in the pediatric department of Taiwan University, School of Medicine, from 347 children who had received blood during surgery, %39/6 showed symptoms suggesting TT virus which had been clearly proved to be in relation with blood transfusions (15). So, to prevent or reduce the aforementioned side effects in general, diagnosing the actual need of patients to blood transfusion is of highest importance.

## Conclusions

According to the results of this study, it can be concluded that the technicians’ performance was primary based on habit and experience. Their knowledge of scientific standards of blood transfusion and blood bag storage conditions were low. These results are serious warnings to the authorities in finding the most appropriate solution for removing this great shortcoming by retraining, conducting training workshops, distributing educational pamphlets and booklets regarding transportation, storage, preparation and standards of blood transfusion for all those who are associated with blood transfusion. Therefore, the error rate is lowered, the knowledge of the technicians is increased, health and safety of patients during anesthesia and after discharge from the hospital are maintained and; thereby, the disability and incidence of incurable diseases in community are prevented while the patients’ rate of health and safety are increased.
